# Advancing the Personalized Advantage Index (PAI): a Systematic Review and Application in Two Large Multi-Site Samples in Anxiety Disorders – ERRATUM

**DOI:** 10.1017/S0033291725000431

**Published:** 2025-05-02

**Authors:** Charlotte Meinke, Silvan Hornstein, Johanna Schmidt, Volker Arolt, Udo Dannlowski, Jürgen Deckert, Katharina Domschke, Lydia Fehm, Thomas Fydrich, Alexander L. Gerlach, Alfons O. Hamm, Ingmar Heinig, Jürgen Hoyer, Tilo Kircher, Katja Koelkebeck, Thomas Lang, Jürgen Margraf, Peter Neudeck, Paul Pauli, Jan Richter, Winfried Rief, Silvia Schneider, Benjamin Straube, Andreas Ströhle, Hans-Ulrich Wittchen, Peter Zwanzger, Henrik Walter, Ulrike Lueken, Andre Pittig, Kevin Hilbert

**Affiliations:** 1 Department of Psychology, Humboldt-Universität zu Berlin, Berlin, Germany; 2 Translational Psychotherapy, Department of Psychology, Friedrich–Alexander University Erlangen–Nürnberg, Erlangen/Nürnberg, Germany; 3 Institute for Translational Psychiatry, University of Münster, Münster, Germany; 4 Department of Psychiatry, Psychosomatics, and Psychotherapy, Center of Mental Health, University Hospital of Würzburg, Würzburg, Germany; 5 Department of Psychiatry and Psychotherapy, Medical Center – University of Freiburg, Faculty of Medicine, University of Freiburg, Freiburg, Germany; 6 German Center for Mental Health (DZPG), partner site Berlin-Potsdam, Germany; 7 Department of Psychology, University of Münster, Münster, Germany; 8 Department of Clinical Psychology and Psychotherapy, Faculty of Psychology, University of Cologne, Cologne, Germany; 9 Department of Biological and Clinical Psychology / Psychotherapy, University of Greifswald, Greifswald, Germany; 10 Institute of Clinical Psychology and Psychotherapy, Technische Universität Dresden, Dresden, Germany; 11 Department of Psychiatry and Psychotherapy & Center for Mind, Brain and Behavior, Philipps-University Marburg, Marburg, Germany; 12 LVR-University Hospital Essen, Department of Psychiatry and Psychotherapy, Faculty of Medicine, University of Duisburg-Essen, Duisburg/Essen, Germany; 13 Center for Translational Neuro- and Behavioral Sciences (CTNBS), University of Duisburg-Essen, Duisburg/Essen, Germany; 14 Social & Decision Sciences, School of Business, Constructor University Bremen, Bremen, Germany; 15 Christoph-Donier Foundation for Clinical Psychology, Marburg, Germany; 16 Mental Health Research and Treatment Center, Ruhr-Universität Bochum, Bochum, Germany; 17 Protect-AD Study Site Cologne, Cologne, Germany; 18 Department of Psychology (Biological Psychology, Clinical Psychology, and Psychotherapy), University of Würzburg, Würzburg, Germany; 19 Department of Experimental Psychopathology, University of Hildesheim, Hildesheim, Germany; 20 Department of Clinical Psychology and Psychotherapy, Faculty of Psychology & Center for Mind, Brain and Behavior, Philipps-University Marburg, Marburg, Germany; 21 Department of Psychiatry and Psychotherapy, Campus Charité Mitte, Charité – Universitätsmedizin Berlin, Berlin, Germany; 22 Department of Psychiatry and Psychotherapy, University Hospital, Ludwig-Maximilians-University Munich, Munich, Germany; 23 kbo-Inn-Salzach-Klinikum, Clinical Center für Psychiatry, Psychotherapy, Geriatrics, Neurology, Gabersee Wasserburg, Germany; 24 Department of Psychiatry and Psychotherapy, CCM, Charité – Universitätsmedizin Berlin, corporate member of FU Berlin and Humboldt Universität zu Berlin, Berlin, Germany; 25 Translational Psychotherapy, Institute of Psychology, University of Göttingen, Göttingen, Germany; 26 Department of Psychology, Health and Medical University Erfurt, Erfurt, Germany

When this article was originally published in Psychological Medicine, Table 1 was incorrectly laid out, with data being used in the wrong columns. This has now been updated online with the correct layout.

The publisher apologises for this error.
